# Transglycosylation Activity of Engineered *Bifidobacterium* Lacto-*N*-Biosidase Mutants at Donor Subsites for Lacto-*N*-Tetraose Synthesis

**DOI:** 10.3390/ijms22063230

**Published:** 2021-03-22

**Authors:** Mireia Castejón-Vilatersana, Magda Faijes, Antoni Planas

**Affiliations:** Laboratory of Biochemistry, Institut Químic de Sarrià, University Ramon Llull, 08017 Barcelona, Spain; mireiacastejonv@iqs.edu

**Keywords:** *Bifidobacterium*, biocatalysis, human milk oligosaccharides, lacto-*N*-biosidase, protein engineering, transglycosylation

## Abstract

The health benefits of human milk oligosaccharides (HMOs) make them attractive targets as supplements for infant formula milks. However, HMO synthesis is still challenging and only two HMOs have been marketed. Engineering glycoside hydrolases into transglycosylases may provide biocatalytic routes to the synthesis of complex oligosaccharides. Lacto-*N*-biosidase from *Bifidobacterium bifidum* (LnbB) is a GH20 enzyme present in the gut microbiota of breast-fed infants that hydrolyzes lacto-*N*-tetraose (LNT), the core structure of the most abundant type I HMOs. Here we report a mutational study in the donor subsites of the substrate binding cleft with the aim of reducing hydrolytic activity and conferring transglycosylation activity for the synthesis of LNT from *p*-nitrophenyl β-lacto-*N*-bioside and lactose. As compared with the wt enzyme with negligible transglycosylation activity, mutants with residual hydrolase activity within 0.05% to 1.6% of the wild-type enzyme result in transglycosylating enzymes with LNT yields in the range of 10–30%. Mutations of Trp394, located in subsite -1 next to the catalytic residues, have a large impact on the transglycosylation/hydrolysis ratio, with W394F being the best mutant as a biocatalyst producing LNT at 32% yield. It is the first reported transglycosylating LnbB enzyme variant, amenable to further engineering for practical enzymatic synthesis of LNT.

## 1. Introduction

Human milk oligosaccharides (HMOs) furnish breast-fed infants with a number of health benefits. Their unique composition, differing from other mammal’s milk, drives active research to synthesize and produce the main HMO structures as supplements for infant formula milks (for recent reviews, see [1,2,3,4,5]). Various beneficial effects are critically important for early development and infant health, functioning as prebiotics and antimicrobial agents as well as exerting immunomodulation effects [6,7]. All HMOs are composed of a lactose unit at the reducing end that is extended with lacto-*N*-biose or *N*-acetyllactosamine through β-1,3 and β-1,6 linkages as linear or branched oligosaccharides to generate at least 13 different core structures from tetra to dodecasaccharides, which are further fucosylated and/or sialylated at different positions. The first two core structures are lacto-*N*-tetraose (LNT, Galβ1,3GlcNAcβ1,3Galβ1,4Glc) core-type 1 and lacto-*N*-neotetraose (LNnT, Galβ1,4GlcNAcβ1,3 Galβ1,4Glc) core-type 2 oligosaccharides [1]. While there are over 150 HMO structures identified, 2’-fucosyllactose (2’-FL) and LNnT are the only commercially available HMOs for supplementing infant formula, produced by fermentation of metabolically engineered *E. coli* strains [2,8]. Since LNT is more abundant in human milk than LNnT (which is mainly found in other mammal’s milks), it is a current target for designing efficient production strategies. Chemical synthesis, enzymatic synthesis (biocatalysis) and fermentation (cell factory) approaches are current focus of research.

LNT production in whole cells was first reported in 2014 with an engineered *E. coli* strain with chromosomal integration of two transferases, the *Neisseria meningitidis* β1,3-*N*-acetylglucosaminyltransferase (LgtA) and *E. coli* β1,3-galactosyltransferase (WbgO) [9], and further improved in antibiotic-free cultures [10]. However, mixtures of lacto-*N*-triose and LNT were produced in these systems, which will require additional metabolic engineering refinement.

LNT chemical and enzymatic syntheses can be grouped in two general strategies: the “linear approach” that mimics the biosynthetic pathway by sequential addition of two monosaccharide units on the non-reducing end of lactose, and the “convergent approach” through coupling of lacto-*N*-biose (LNB) and lactose as disaccharide building blocks. Recent chemical synthesis of LNT includes both strategies, the linear [11] and convergent [12] approaches, where the protecting-leaving group combinations were critical to achieve successful glycosylations. The low yields and multistep pathways currently limit the scalability of the chemical synthesis. 

To overcome the limitations of chemical synthesis and develop green and sustainable technologies, current efforts are addressed to enzymatic synthesis, with two competing strategies: the use of hydrolytic glycosidases endowed with some natural transglycosylating activity but often requiring further enzyme engineering to improve the transglycosylation over hydrolysis ratio, and the use of glycosyltransferases with sugar nucleotide donors, which are highly efficient but often difficult to isolate and require expensive donors or complex regeneration systems.

With glycosyltransferases mimicking the biosynthetic pathway, GlcNAc transfer from UDPGlcNAc to lactose catalyzed by *N. meningitidis* LgtA produced lacto-*N*-triose at 87% yield [13]. Next, galactosyl transfer from UDPGal using the β-1,3-galactosyltransferase WbgO from *E. coli* afforded benzyl-LNT at 87% at the mg scale [14]. An improved strategy has been recently reported by the Chen group using two sequential one-pot multienzyme (OPME) systems [15]. Thanks to the identification of a highly efficient β-1,3-galactosyltransferase from *Chromobacterium violaceum* (*Cv*β3GalT), multigram scale synthesis of LNT at 99% yield from lactose was achieved. Although highly efficient, the use of glycosyltransferases for preparative and large scale synthesis is hampered by the need for expensive sugar nucleotide donors. Glycosyl hydrolases able to catalyze the synthesis of glycosidic bonds by kinetically-controlled transglycosylation from cheap and stable arylglycoside donor substrates are an attractive alternative for industrial applications, but such an approach is not yet well developed for LNT synthesis.

Grounded on the “linear approach” starting from lactose, a transglycosylating mutant of *Bifidobacterium bifidum N*-acetylhexosaminidase (*Bb*H1) has been recently reported to give 84% yield in lacto-*N*-triose using GlcNAc-*p*NP (*p*NP: *p*-nitrophenyl) as donor substrate [16]. However, elongation with *Bacillus circulans* β-galactosidase-catalyzed transglycosylation of *o*-nitrophenyl β-galactoside to lacto-*N*-triose rendered a poor 22% yield in LNT [17,18]. With regard to the “convergent approach”, direct coupling of lacto-*N*-biose (LNB) to lactose through a β-1,3 linkage has been attempted by transglycosylation reactions using lacto-*N*-biosidases. The first lacto-*N*-biosidase-mediated transglycosylation used an enzyme from *Aureobacterium* sp., although it was rather inefficient, with LNT yields of 1-3% [17]. When lacto-*N*-biosidase was discovered from *B. bifidum* (LnbB) showing its metabolic function to hydrolyze LNT to LNB and lactose for uptake by bifidobacteria, the authors also detected some transglycosylation activity when using LNB-*p*NP and lactose as donor and acceptor substrates [19]. A different strategy based on the glycosynthase-like technology has been recently reported by the Nidetzky group using a mutant with compromised hydrolase activity and lacto-*N*-biose 1,2-oxazoline as activated donor substrate [20]. Whereas the mutant D320A showed very low synthase activity, the D320E mutant produced LNT up to 30% yield, which was stable over extended reaction times, but the enzyme still retained high activity for hydrolysis of the intrinsically labile oxazoline donor.

**Figure 1:**
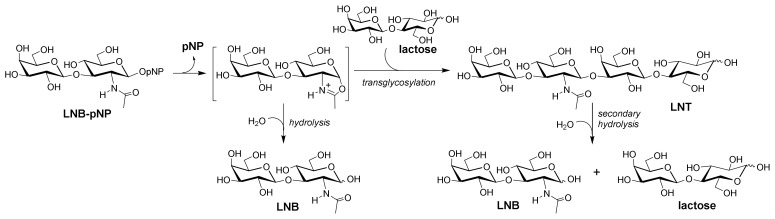
Synthesis of lacto-*N*-tetraose (LNT) by transglycosylation with engineered lacto-*N*-biosidase (LnbB) variants. Hydrolysis: primary hydrolysis of the donor substrate; secondary hydrolysis: hydrolysis of the product.

The originally detected transglycosylation activity of LnbB [19] prompted us to initiate a protein engineering program to enhance the synthetic capabilities of the enzyme with a stable arylglycoside donor substrate amenable for scaling up LNT synthesis. Here we explore the ability of LnbB mutants to perform direct transglycosylation reactions using LNB-*p*NP as donor substrate (Figure 1). Based on the crystal structure of *LnbB* in complexes with LNB and LNB-thiazoline [21] and our previous study on the conservation of active site topologies in family GH20 (*N*-acetylhexosaminidases including LnbB) [22], a series of donor subsite mutants were designed and evaluated for their transglycosylation activity and diminished hydrolase activity. We report the first transglycosylating LnbB variant for LNT synthesis.

## 2. Results

### 2.1. LnbB Mutants Design

*Bifidobacterium bifidum* LnbB [19,22] is a member of family GH20 in the Carbohydrate Active Enzymes (CAZy) classification [23] that operates by substrate-assisted catalysis. The 2-acetamido group of the substrate acts as an intramolecular nucleophile leading to an oxazoline/oxazolonium intermediate that is attacked by a nucleophilic water to render the final hydrolysis product with net retention of the anomeric configuration [24,25,26] (Figure 1, detailed mechanism in Figure S1, Supplementary Materials). In retaining glycoside hydrolases, transglycosylation (T) takes place in competition to hydrolysis (H), and a number of studies on diverse GH families are addressing different strategies to modify the T/H ratio. Not only the donor substrate but also the transglycosylation product can be hydrolyzed (secondary hydrolysis) as it is the natural substrate of the hydrolytic activity of the enzyme, thus requiring tight kinetic control. Protein engineering approaches to modulate the T/H ratio towards improved transglycosylation can be grouped in three complementary strategies (reviewed in [3,27]): (i) decrease hydrolysis by mutations at the donor (negative) subsites of the active site that reduce transition state stabilization, (ii) enhance acceptor binding to compete with water by mutations that increase affinity to the acceptor (positive) subsites, and (iii) modify the hydrophilic/hydrophobic balance at the active site to reduce the accessibility or reactivity of the nucleophilic water. A promising approach towards reducing the hydrolase activity while enhancing transglycosylation (strategy (i) above) is based on replacing the enzyme’s most conserved residues by structural analogues in the donor subsites [28].

**Figure 2:**
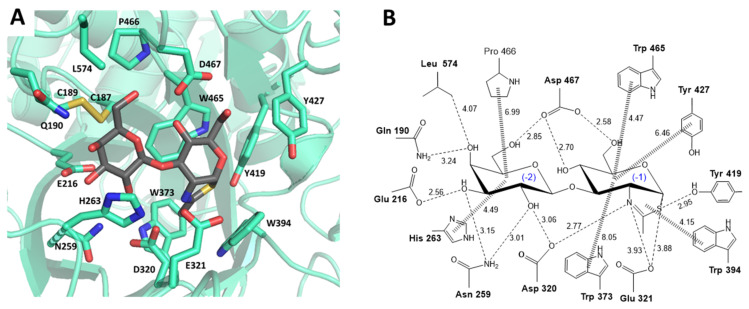
(**A**) X-ray structure of *Bifidobacterium bifidum* lacto-*N*-biosidase (LnbB) complexed with LNB-thiazoline inhibitor (PDB ID: 4JAW). First shell residues shaping the donor subsites in the active site are shown in sticks. (**B**) Enzyme-ligand interactions map at donor subsites (-2, -1). Amino acid residues mutated in this work are in bold.

Inspection of the X-ray structure of LnbB in complex with LNB and LNB-thiazoline [21] identifies the amino acid residues that directly interact with the ligand in the donor subsites (-2 and -1) (Figure 2). Most of them are highly conserved among GH20 enzymes (see sequence alignment in Figure S2) and were selected for mutational analysis. 

First shell residues in subsite -1, Trp373, Trp394, Tyr419 and Trp465 are conserved in GH20 enzymes and were replaced by Phe. Other mutations for Trp394 were also analyzed since it is located in a position not only interacting with the substrate but also close to the catalytic acid/base Glu321. Tyr427, although not conserved, is also in the first shell and was mutated to Phe. Subsite -2 is dominated by hydrogen-bonding interactions with the substrate, and both conserved (Asp320, Asp467, Asn259 and His263) and non-conserved (Gln190, Glu216 and Leu 574) residues were replaced by structural analogues or alanine (Table 1). The residues of the catalytic machinery, the acid/base Glu321 and Asp320 as assisting residue, were also mutated as controls to evaluate the residual hydrolase activity due to knocking out these essential residues.

### 2.2. Hydrolase Activity of LnbB Mutants

To evaluate the impact of the mutations on the hydrolytic activity of the enzyme, specific activities (SA_H_) with 250 μM LNB-*p*NP substrate at pH 4.5 (optimum pH of the wt enzyme [22]) and 30 ºC were determined by monitoring the release of *p*NP (discontinuous assay, see Methods). Under these conditions of substrate concentration below *K*_M_ (≈ 500 μM for the wt enzyme, see below), SA_H_ reflects *k*_cat_/*K*_M_ in order to compare the effect of the mutations on catalytic efficiency. As summarized in Table 1, all mutations decreased the hydrolase activity, with residual activities ranging from 60 to 0.1% relative to the wild type enzyme, except the mutant D320A at the catalytic assisting residue that was highly inactive and retained less than 0.04% of the wt activity (10^4^-fold decrease), and W394K that was inactive probably due to the destabilizing positive charge introduced close to the anomeric carbon (strongly destabilizing the oxocarbenium-like transition state). In general, mutations at residues located in the -1 subsite had a larger impact than those located in subsite-2.

### 2.3. Formation of LNT by Transglycosylating LnbB Mutants

**Figure 3:**
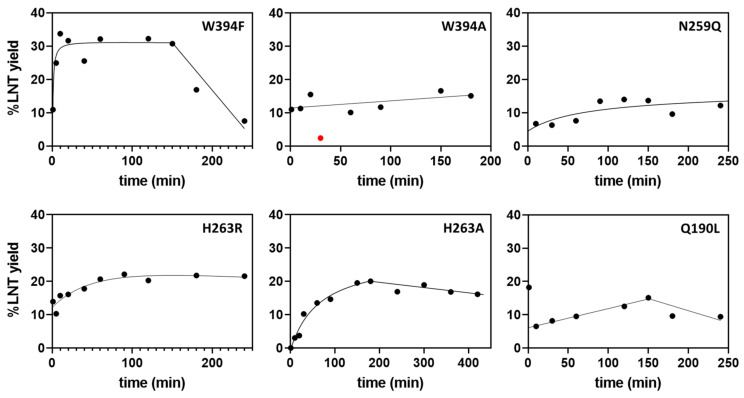
Time course monitoring of LNT formation by HPLC-MS for selected mutants (%LNT max >15%).

Wild-type LnbB has negligible transglycosidase activity [19]. The ability of the mutant enzymes to perform transglycosylation was first assayed by monitoring the time course of LNT product formation (by HPLC-MS, see methods) using 2.5 mM LNB-*p*NP donor and 200 mM lactose acceptor at pH 4.5 and 30 °C, corresponding to saturating donor concentration and high acceptor/donor ratio. Results are summarized in Table 1 and expressed as maximum yield in LNT product (%LNT). For those mutants showing transglycosylation, LNT is initially formed to reach a maximum value, but then it is hydrolyzed due to secondary hydrolysis. Figure 3 presents the time course of LNT formation of representative transglycosylating mutants. Two features are considered in these plots: the maximum LNT yield and the time interval in which the product lasts before it is hydrolyzed.

**Figure 4:**
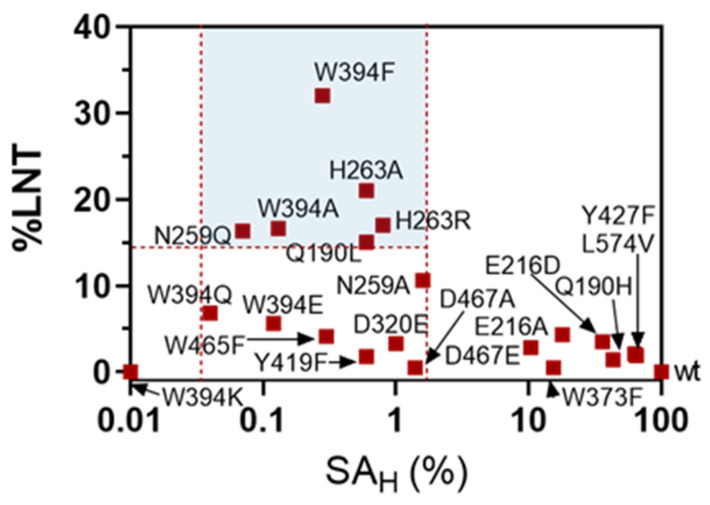
Transglycosylation yields (%LNT) *vs.* hydrolase activity (SH_A_) plot for LnbB mutants at donor subsites. Transglycosylating mutants are those with a SA_H_ between 0.05 and 1.6 % of the wt enzyme.

When plotting LNT maximum yield of the transglycosylation reaction (%LNT) against hydrolase activity on LNB-*p*NP (SA_H_ in the absence of acceptor), the different mutants can be clustered in three groups (Figure 4): mutant enzymes with residual hydrolase activity (SA_H_) >1.6% of the wt, which do not have (or have low) transglycosylation activity, highly inactive mutants with SA_H_ <0.05% of the wt, and mutants that lay in between, with 0.05% < SA_H_ < 1.6%, which present significant transglycosylation activity. In this last group, six mutants show a maximum LNT yield higher than 15% (W394F, W394A, Q190L, N259Q, H263R, H263A), highlighting the role of these residues in the donor subsites, Trp394 in subsite -1, and Gln190, Asn259 and His263 in subsite -2, whose mutation strongly reduces the hydrolase activity and enhances the transglycosylation capability in the presence of acceptor. Larger enzyme inactivation, as in the mutants with SA_H_ lower than 0.05% of the wt enzyme, leads to essentially dead enzymes unable to catalyze neither hydrolysis nor transglycosylation. Therefore, a window of strongly reduced but not abolished hydrolase activity (in the range of 0.05 to 1.6% of the wt activity) seems to furnish the mutant with transglycosylating activity. Of all mutants, W394F was the most promising with 32% yield in LNT formation and a significant life-time of the product before secondary hydrolysis became significant (Figure 3).

Reactions were performed at pH 4.5, that is, the pH optimum of the hydrolase activity of the wt enzyme [22]. To evaluate the effect of pH on the transglycosylation activity, the two best mutants in terms of %LNT (W394F and H263A) were analyzed at higher pH values with the aim of reducing the hydrolase activity that may result in increased transglycosylation yield. However, no significant effect was observed in the range of pH 4.5 to 7, and a decrease in %LNT at pH 9 (data not shown).

When combining the best transglycosylating single mutants (at positions Trp394, His263 and Gln190) with other mutations with significant activity (Table 2), none of them gave higher %LNT than the parental single mutants. Therefore, no synergistic effects were observed with these combinations.

### 2.4. Transglycosylation Activity of Selected Mutants

Mutants showing a maximum LNT yield >15% were selected to determine the specific activity in transglycosylation mode (SA_T_) and calculate an apparent T/H ratio. Release of *p*NP relates to the first step in the double displacement mechanism and therefore the specific activities calculated from the rate of *p*NP release are an aggregate value that accounts for the global activity. In the absence of an acceptor, SA_H_ is the hydrolase activity (since no self-condensation occurs due to the substrate specificity of the enzyme), whereas in the presence of an acceptor the initial rate of *p*NP release reflects transglycosylation but also includes donor hydrolysis at initial reaction times (SA_T_). Using a constant donor concentration (250 μM LNB-*p*NP, not saturating concentration), initial rates were determined in the absence and presence of lactose acceptor (concentration varying from 100 to 600 mM) (Figure S3). For W394F, H263R and N259Q rates increased with lactose concentration up to a maximum at 200–400 mM lactose, whereas for mutants W394A and Q160L, the opposite effect was observed (inhibition of *p*NP release by lactose) and H263A showed essentially no dependence with lactose concentration. SA_T_ was calculated at 200 mM lactose in order to compare the apparent T/H ratio among mutants. 

Initial rates of hydrolysis (SA_H_, in the absence of acceptor) and transglycosylation at 200 mM lactose (SA_T_) are summarized in Table 3. Mutant W394F displayed the highest SA_T_/SA_H_ ratio (1.41), consistent with its higher maximum yield (32%) in LNT formation (%LNT, Table 1), followed by N259Q and H263R. For the other mutants, no correlation between the apparent T/H ratio and %LNT was observed since all of them rendered essentially the same maximum yield of LNT (%LNT in the range of 15 to 21%).

### 2.5. Kinetic Parameters for Donor Substrate in Hydrolysis and Transglycosylation Modes

To further understand the effect of the mutations at the donor subsites on the apparent T/H ratio, the kinetic parameters for hydrolysis and transglycosylation reactions of the selected mutants were evaluated. Donor kinetic constants were determined from the initial rates of *p*NP release when varying the donor concentration (1 to 1000 μM LNB-*p*NP) in the absence of an acceptor (hydrolase activity) and at fixed saturation concentration of an acceptor (200 mM lactose). Kinetics followed a hyperbolic dependence with the donor substrate and data were fitted to a Michaelis-Menten equation to calculate the apparent kinetic constants (Table 4 and Figure S5).

Mutants H263R and Q190L still retain significant residual hydrolase activity, with *k*_cat_/*K*_M_ values of 2-orders of magnitude lower than the wt enzyme, whereas the other mutants (including the best transglycosylating mutant W394F) showed a 3-orders of magnitude reduction of hydrolase activity. In the presence of a lactose acceptor, a significant increase of apparent *K*_M_ of the donor is only observed for W394A (about 6-fold), whereas the other mutants show a minor effect of lactose on apparent *K*_M_ values (≤2-fold) for the donor substrate. Significantly, the apparent *K*_M_ for the best transglycosylating mutant (W394F) is not altered but *k*_cat_ (for *p*NP release) is doubled in the presence of lactose, consistent with its higher yield in LNT product. Since *K*_M_ values are different among mutants, the apparent T/H ratios were recalculated from *k*_cat_/*k*_M_ values (Table 4, Figure S4), but they followed the same trend as the apparent T/H ratios at 250 μM donor concentration shown in Table 3. These data also confirm that transglycosylation reactions of all selected TG mutants at 2.5 mM donor, 200 mM acceptor to determine %LNT (Table 1) were at saturating conditions of the donor substrate.

### 2.6. Regioselectivity of Tetrasaccharide Formation by LnbB W394F Mutant

**Figure 5:**
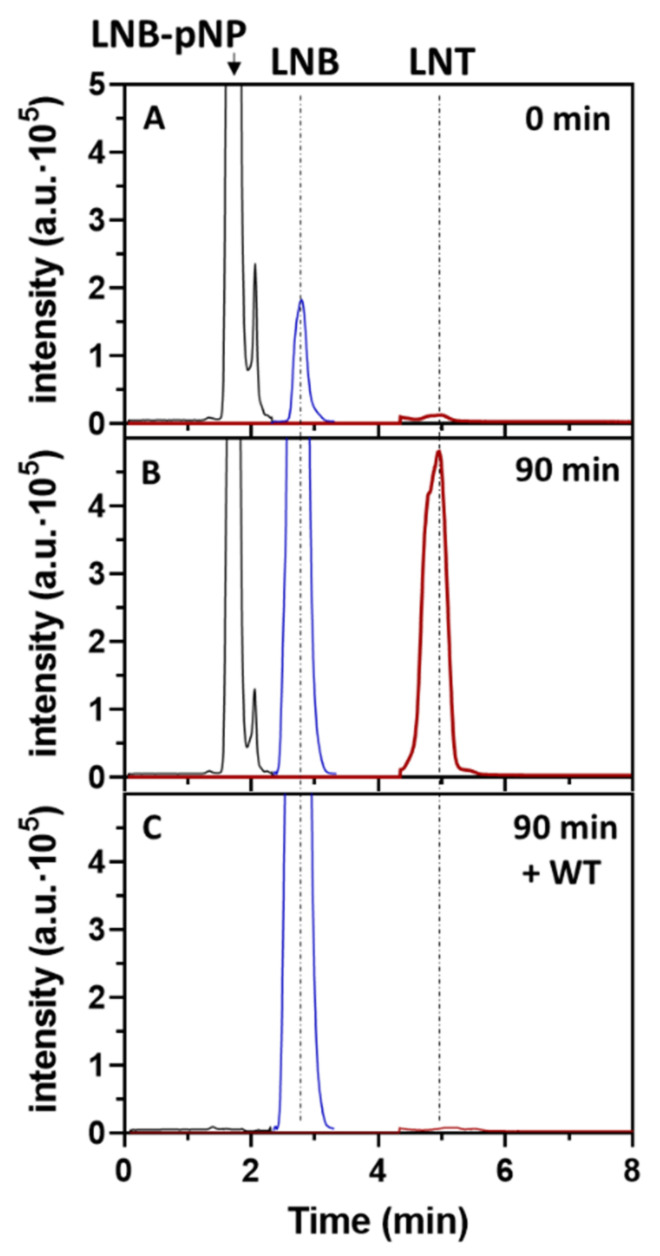
HPLC-MS monitoring of the transglycosylation reaction catalyzed by LnbB W394F. Reac- tion conditions: 1 μM enzyme, 2.5 mM LNB-*p*NP, 200 mM lactose, citrate-phosphate buffer pH 4.5. (**A**) t = 0 min; (**B**) t = 90 min, followed by heat inactivation; (**C**) After addition of wt LnbB (1 μM) for 15 min. Detection by EI-MS. Lactose acceptor is not shown since it is in large excess.

Mutant W394F is the best transglycosylating mutant to synthetize LNT. The protein is produced with similar expression yields to the wt enzyme and has the same thermal stability (Figure S6). To assess the regioselectivity of glycoside bond formation, the reaction product was treated with wt LnbB to monitor hydrolysis. As shown in Figure 5, a standard reaction with 2.5 mM LNB-*p*NP + 200 mM lactose and 1 μM W394F enzyme was left until the product was accumulated, followed by enzyme heat inactivation at 100 ºC. Then, wt enzyme was added, showing a rapid hydrolysis of the product back to LNB and lactose. The remaining LNB-*p*NP donor still present in the heat-inactivated reaction was also immediately hydrolyzed by the wt enzyme. Since wt LnbB is specific for hydrolysis of the central β1,3 linkage of LNT (inactive on a β1,4 linkage [19]) and the retention time of the transglycosylation product is identical to that of a LNT standard, these results are in agreement with β1,3 glycosidic bond formation in the TG reaction. 

## 3. Discussion

With the aim of engineering TG activity into LnbB, selection of donor subsite residues was based on the emerging concept that mutation of conserved residues interacting with the substrate may unbalance the T/H ratio towards transglycosylation [28]. We selected residues located in subsites -1 and -2 (donor subsites) of LnbB that interact with the substrate according to the X-ray structure of an enzyme-ligand complex (Figure 2, [21]), most of them conserved within family GH20 *N*-acetylhexosaminidases. Destabilizing enzyme-donor interactions at the transition state will reduce hydrolytic activity but eventually improve TG activity. Similar mutations have been analyzed in few related GH20 enzymes. To compare results, targeted residues can be grouped as follows:

### 3.1. Assisting Residue

In LnbB, mutation D320A has a drastic effect on hydrolase activity, resulting in an essentially dead enzyme, whereas D320E has a significant effect (1% hydrolase activity) but induces poor TG activity (3.3% LNT, Table 1). Equivalent mutations in GH20 *N*-acetylhexosaminidases showed different results. *B. bifidus Bb*H1 has a quite high native TG activity (45% in [16], 16% in [28] depending on the experimental conditions) to produce lacto-*N*-triose (LNT-II) from GlcNAc-*p*NP and lactose as donor and acceptor substrates. Upon mutation of the assisting residue, D746T (also labeled as D714T depending on the sequence numbering) exhibited improved TG activity with 84.4% yield in LNT-II [16]. Likewise, mutations of the assisting residue in *T. flavus Tf*Hex (D370G, A, L or N) showed a large decrease of hydrolase activity but, surprisingly, D370V had higher activity. Those mutants with reduced hydrolase activity all presented some TG activity with GlcNAc-*p*NP as a donor and acceptor substrate to produce a mixture of chitooligosaccharides [29]. From the results reported here, LnbB mutants at the assisting residue strongly reduce the hydrolytic activity but, as opposed to other GH20 enzymes, have no impact on TG activity. Assisting residue mutations have also been assayed in glycosynthase-type reactions using oxazoline donors. In *Bb*H1 with GlcNAc-oxazoline as activated donor and lactose acceptor, LNT-II was produced in 86% yield with mutant D746E [30]. The yield was further improved with an immobilized version of the enzyme packed into a fixed bed for continuous production of LNT II [31]. The high yields with the *Bb*H1 enzyme mutants both with GlcNAc-*p*NP and GlcNAc-oxazoline donors do not translate to LnbB. With LnbB D320E using LNB-oxazoline as donor and lactose as an acceptor, LNT product reached a significantly lower 30% yield due to the high hydrolysis of the oxazoline donor [20] and, as shown here, the same mutant with LNB-*p*NP donor showed poor TG activity.

### 3.2. Hydrophobic Platform

Three conserved tryptophan residues (Trp373, Trp394 and Trp465) in subsite -1 of LnbB, when mutated to reduce the hydrolase activity, have different impacts on TG activity (Table 2). Trp394 and Trp465 are both located in subsite -1. Trp465 establishes a stacking interaction with the GlcNAc ring whereas Trp394 is perpendicular to the sugar ring and close to C1 and C2, stabilizing the oxazoline/oxazolonium intermediate (Figure 2). W465F mutant reduces the hydrolase activity (0.3% of the wt) but has minor effect on TG activity (4.1% yield in LNT, Table 1). Remarkable is the W394F mutant, with 0.3% hydrolase activity but with outstanding TG activity, reaching 32% LNT yield. An Ala mutation (W394A) also showed a significant TG activity (16.6% LNT yield), while polar mutations in this position (W394K, E, Q) were detrimental (Table 1). Conserved Trp residues in other GH20 *N*-acetylhexosaminidases also impact the T/H ratio. In *Bb*H1, mutation W882H (position equivalent to Trp465 in LnbB) significantly improved TG activity with 66% yield in LNT-II formation (compared to 16% for the WT), whereas W801H (position equivalent to Trp394 in LnbB) retained the same TG activity as the WT (17%). These results are opposed to those obtained here for LnbB mutants, since W394F in LnbB gave the highest TG yield while mutation of the equivalent position in *Bh*H1 (W801H) had no effect on TG activity, and mutant *Bh*H1 W882H gave high TG activity whereas the equivalent LnbB residue (W465F) had a minor effect on TG activity. Although equivalent positions in the sequence alignment, these results may reflect the different relative orientation of the side chains in the 3D structures (not solved for *Bh*H1). Finally, Trp373 in LnbB was also mutated, but to the best of our knowledge, the equivalent position has not been studied in other GH20 enzymes [32]. Although close to the sugar ring in subsite -1, W373F retained 15% of the wt hydrolase activity and had almost no effect on TG activity. The Phe substitution seems to maintain the stacking interaction with little effect on activity. 

### 3.3. Catalytic Tyrosine

Tyr419 in LnbB is a conserved essential residue in GH20 enzymes as well as in GH18 chitinases and GH85 endo-β-*N*-acetylglucosaminidases, which is located in subsite -1 interacting with the oxazoline intermediate [24,25]. Mutation of this Tyr residue in *Tf*Hex (Y470F, H, N) drastically reduced the hydrolase activity (0.5 to 4% of the wt) but conferred TG activity with GlcNAc-*p*NP as substrate leading to chitooligosaccharides with moderate yields [33]. In *Bh*H1, mutant Y827F was used in glycosynthase-type reactions with GlcNAc-oxazoline donor and lactose as an acceptor reaching up to 80% yield in LNT-II at a short reaction time, but the product was degraded by the residual hydrolase activity of the enzyme [30]. However, the equivalent mutation Y419F in LnbB, although it only retained 0.5% hydrolase activity, showed low (1.8%) TG activity. This mutant was also tested in a glycosynthase-type reaction with LNB-oxazoline, but the residual hydrolase activity rapidly degraded the product [20]. 

### 3.4. Mutations in Subsite -2

We also evaluated the effect of mutations at residues interacting with the donor substrate in subsite -2 that have no equivalent in *N*-acetylhexosaminidases where the binding cleft is restricted to subsite -1. Residues Gln190, Glu216, Asn259, His263, Asp467 and Leu574 shape subsite -2 for accommodation of the β1,3-linked Gal unit of the substrate by establishing a network of H-bonding interactions with all the hydroxyl groups of the substrate. Mutants that reduce the hydrolase activity to about 1% than that of the wt (Q190L, N259A,Q; H263A,R, Table 1) do show TG activity with LNT yields in the range of 10-20%. Asp467 is an exception; it interacts with both sugar units in subsites -1 and -2 but D467A, with 1.4% hydrolase activity, is not a transglycosylating mutant. This is in agreement with mutation of the equivalent residue Asp884 of *Bh*H1, where the D884N mutant even reduced the wt TG activity (12% for D884N compared to 16% for wt) [28].

## 4. Materials and Methods

### 4.1. Substrates

*p*-Nitrophenyl lacto-*N*-bioside (LNB-*p*NP) and lacto-*N*-tetraose (LNT) were obtained from Biosynth Carbosynth. Lacto-*N*-biose (LNB) was kindly provided by Dr. M. Kitaoka (National Agriculture and Food Research Organization, NARO, Tsukuba, Ibaraki, Japan). Lactose and all general chemicals were from Sigma-Aldrich.

### 4.2. Mutants Preparation by Site Directed Mutagenesis

The full-length LnbB gene was previously cloned into a pET24b vector (Novagen, Madison, WI, USA) obtaining the expression plasmid pET24b-LnbB-FL [22]. This construct was used as template to introduce single and double mutants by PCR Site-Directed Mutagenesis (Quik Change protocol, Agilent Technologies, Santa Clara, CA, USA). For each single mutant, a sense/antisense partially overlapping primers pair was designed (Table S1). Double mutants were prepared on the previous single mutant as template in a second mutagenesis reaction. The PCR reactions were performed with Iproof polymerase in a final volume of 40 µL containing 5 ng of template DNA, 0.5 µM of each sense/antisense primer and Iproof High-fidelity 2× mix (0.04U/µL, Bio-Rad). PCR conditions consisted of an initial denaturalization (98 °C for 3 min) followed by 30 cycles of denaturalization (95 °C for 30 s), annealing (30 s at different annealing temperatures depending of the primer), extension (72 °C for 4 min), and final extension (72 °C for 3 min). PCR products were purified with GenElute™ PCR Clean-Up Kit from Sigma-Aldrich, St. Louis, MO, USA, then digested with *Dpn*I during 1.5–3 h at 37 °C. After enzyme inactivation at 80 °C for 20 min, *E. coli* DH5α competent cells were transformed with the mutagenesis reactions. Isolated plasmids from the transformants were verified by DNA sequencing (Stab Vida, Caparica, Portugal) and used to transform *E. coli* BL21 (DE3) Star cells for protein expression.

### 4.3. Expression and Purification of Lacto-N-Biosidase Mutants

*E. coli* BL21 (DE3) Star cells harboring the mutated pET24b-Lnb-FL plasmids were grown following an autoinduction protocol [34] in Luria Broth (LB) media supplemented with kanamycin (30 µg/mL), Na_2_HPO_4_ (25 mM), K_2_HPO_4_ (25 mM), NH_4_Cl (50 mM), Na_2_SO_4_ (5 mM), MgSO_4_ (2 mM), glycerol (0.5% *w/v*), glucose (0.05% *w/v*) and lactose (0.2% *w/v*) in a 30 mL final volume. Cultures were grown at 250 rpm, 30 °C for 24 h.

Cells were harvested by centrifugation and resuspended with 800 µL of resuspension buffer (20 mM Na_2_HPO_4_, 150 mM NaCl, pH 7.5) supplemented with 1 mM of PMSF (phenylmethylsulfonyl fluoride). Cells were disrupted by sonication in a Soniprep 150 sonifier at 4 °C (7 min, 10 s ON/ 20 s OFF, 50% amplitude); the soluble fraction was separated by centrifugation (14,000 rpm, 30 min, 4 °C). Proteins were purified using His SpinTrap columns (immobilized metal ion affinity chromatography) (GE Healthcare) following the manufacture’s guidelines. Proteins were eluted with 50 mM imidazole in 20 mM Na_2_HPO_4_, 150 mM NaCl, pH 7.5. The buffer was exchanged to resuspension buffer (20 mM Na_2_HPO_4_, 300 mM NaCl at pH 7.5) using a 30kDa Amicon ultrafiltration unit (Merk, Kenilworth, NJ, USA). Protein concentrations were determined with BCA Protein Assay Kit (ThermoFisher, Waltham, MA, USA). 

### 4.4. Hydrolase Activity Assay

The enzymatic reactions were performed at 30 °C in a total volume of 250 µL. The enzyme (different concentrations depending of the mutant) in 50 mM citrate-phosphate buffer at pH 4.5 was pre-incubated for 5 min at 30 °C. Then the reaction was initiated by adding LNB-*p*NP to 250 µM. At regular time intervals, 20 µL of reaction were added to 150 µL stop buffer (500 mM glycine at pH 11) using a Bravo liquid handling Robot (Agilent Technologies, Santa Clara, CA, USA). The released *p*NP was measured at 405 nM in a microplate reader BioTec EL × 808. Chromophore concentrations were calculated by interpolation to a standard curve in the same buffer (1.3–300 µM *p*NP).

### 4.5. Transglycosylation Activity Assay

Transglycosylation reactions were done using 1 μM enzyme, 2.5 mM LNB-*p*NP as a donor and 200 mM lactose as an acceptor in 50 mM citrate/50 mM phosphate buffer at pH 4.5 (or buffer at pH 4.5 to 9 for pH studies) in a total volume of 250 µL. All components except enzyme were pre-incubated at 30 °C for 5 min and the reaction started by adding the enzyme. At different time intervals 20 µL of reaction were extracted and mixed with 60 µL of stop solution (propanol-H_2_O (1:1)) and stored at 4 °C. Samples were analyzed by HPLC-MS (Infinity 1260 HPLC, ESI-MS 6100 series SQ, Agilent Technologies, Santa Clara, CA, USA) using ACQUITY UPLC BEH Amide column 130 Å, 1.7 µm 2.1 × 100 mm (Waters) coupled to ACQUITY UPLC^®^ BEH Amide VanGuard Pre-column 130 Å, 1.7 µm 2.1 × 5 mm (Waters) with 3 µL injection and isocratic elution with acetonitrile/water (65:35), 1% formic acid at 40 °C at a flow rate of 0.2 mL/min. MS detection was done in SIM mode monitoring different ion masses: [LNB-*p*NP + NH_4_]^+^ and [LNB-*p*NP + Na]^+^ (*m/z* 522 and 527); [LNB + H]^+^ and [LNB + Na]^+^ (*m/z* 384 and 406); [LNT + H]^+^ and [LNT + Na]^+^ (*m/z* 708 and 730). External standards were used to quantify LNB, LNB-*p*NP and LNT.

### 4.6. Enzyme Kinetics

Kinetics were carried out at 30 °C in a final volume of 250 µL using a Bravo liquid handling Robot (Agilent, Santa Clara, CA, USA). Enzymatic reactions were done with different LNB-*p*NP concentrations (between 0 and 1 mM) and lactose (0 to 600 mM) in 50 mM citrate / 50 mM phosphate buffer, pH 4.5. Solutions were pre-incubated at 30 °C for 5 min, then the enzyme was added to give a final concentration ranging from 10 nM to 1 μM depending on the mutant. At different time intervals, 20 µL were withdrawn and mixed with 150 µL of stop buffer (0.5 M of glycine pH 11). The *p*-nitrophenol released was detected by absorbance at 405 nm in a microplate reader BioTec ELx808. *p*-Nitrophenol standard curve was performed under the same reaction conditions.

### 4.7. W394F Thermal Stability

Purified protein (W394F or wt LnbB; 4 μM) in PBS (50 mM phosphate, 300 mM NaCl, pH 7.5) was mixed with the commercial dye Sypro Orange (Thermo Fisher Scientific, Waltham, MA, USA; 1:5000 dilution according to the manufacturer’s protocol) in a final volume of 25 μl. The samples were subjected to a thermal gradient in a thermocycler (Rotogene 3000, Corbett Research, Cambridge, UK), consisting of 1 min at 25 °C followed by 1 °C increments (30 s at each temperature) up to 95 °C. The fluorescence was measured (ex 483 nm, em 560 nm), and Tm was determined by fitting the data to a Boltzmann sigmoidal equation using Prism (Prism v.8.3.0 Software, GraphPad, San Diego, CA, USA).

### 4.8. Regioselectivity of Glycoside Bond Formation by W394A

A solution of donor and acceptor substrates in buffer at pH 4.5 was incubated at 30 °C for 5 min and LnbB W394F enzyme was added to a final reaction volume of 150 µL with final concentrations: 2.5 mM LNB-*p*NP, 200 mM lactose and 1 µM W394F in 50 mM citrate-phosphate buffer at pH 4.5. At different time intervals (1, 30, 60 and 90 min), 10 µL aliquots were withdrawn and mixed with 30 µL of stop solution propanol-H_2_O (1:1) stored at 4 °C. Samples were analyzed by HPLC-MS (see transglycosylation assay method section). After 90 min, the enzyme was inactivated by heating at 100 °C for 10 min and the reaction was analyzed by HPLC-MS following the same procedure. Then, to 30 µL of the inactivated reaction at 30 °C, 2 µL of WT enzyme (71.8 µM stock) were added. After 5 and 15 min, 10 µL aliquots were withdrawn, mixed with 30 µL of stop solution and analyzed by HPLC-MS (SIM mode): LNB-*p*NP (donor substrate) at [M+NH_4_]^+^ (*m/z* 522) + [M+Na]^+^ (*m/z* 527); LNB (hydrolysis product) at [LNB+H]^+^ (*m/z* 384) + [LNB+Na]^+^ (*m/z* 406); LNT (TG product) at [M+H]^+^ (*m/z* 708) + [M+Na]^+^ (*m/z* 730).

## 5. Conclusions

The comparative analysis of different GH20 mutant enzymes indicates that the background TG activity already present in the parental wt enzymes correlates with the improvement that can be achieved by the targeted mutations. Wt *Bh*H1 and *Tf*Hex *N*-acetylhexosaminidase already possess some TG activity and the selected mutations (assisting residue, conserved Trps and catalytic Tyr) result in a significant improvement of transglycosylation activity. On the other hand, wt LnbB has negligible TG activity (detected in [19] and in this work, but below the quantification limit) and most targeted mutations only afford low LNT product yields. As shown here, only mutants whose residual hydrolase activity is in the range of 0.05 to 1.6% of the wt enzyme acquire some TG activity, with LNT maximum yields of 15–30%. In this framework, the W394F mutant is outstanding since this single mutation confers TG activity of up to 32% yield in LNT product. Compared with reported glycosynthase-type mutants with the labile LNB-oxazoline donor, the transglycosylating W394F mutant has the advantage of using a stable *p*NP-glycoside as donor substrate and therefore the reaction is amenable to be scaled up. A follow-up of this work will be the introduction of additional mutations in the acceptor subsites with the goal of adding beneficial binding interactions for the lactose acceptor that may result in even further enhanced TG efficiency.

## Figures and Tables

**Table 1:** Hydrolase activity on LNB-*p*NP and transglycosylation yields for LNT by LnbB mutants.

RESIDUE (Subsite)	MUTANT	SA_H_^1^ (s^−1^)	%H ^2^ (mut/wt)	% LNT ^3^ Yield (max)	t (min) ^4^ Max Yield	t Interval ^5^ (min)
WT	WT	24 ± 1.8	100	n.d.^6^		
Trp373 (-1)	W373F	3.7 ± 0.2	15.4	0.5	30	30–120
Trp394 (-1)	W394F	0.07 ± 0.01	0.28	32	105	60–150
	W394A	0.030 ± 0.001	0.13	16.6	150	90–180
	W394K	<0.01	<0.04	n.d.		
	W394E	0.031 ± 0.002	0.12	5.6	180	20–360
	W394Q	0.011 ± 0.001	0.04	6.8	1440	1434–45
Tyr419 (-1)	Y419F	0.13 ± 0.09	0.6	1.8	1	1–10
Tyr427 (-1)	Y427F	15.4 ± 0.8	64	2.1	1	1–3
Trp465 (-1)	W465F	0.06 ± 0.03	0.3	4.1	300	1–360
Glu321 *(-1)	E321A	0.80 ± 0.08	3	-		
Asp320 ** (-1/-2)	D320A	<0.01	<0.04			
	D320E	0.24 ± 0.02	1.0	3.3	90	60–120
Gln190 (-2)	Q190L	0.14 ± 0.02	0.6	15	150	120–180
	Q190H	10 ± 1	43.3	1.4	240	1–240
Glu216 (-2)	E216A	4.3 ± 0.2	18.0	4.3	5	1–10
	E216D	8.7 ± 0.5	36	3.5	10	1–60
Asn259 (-2)	N259A	0.4 ± 0.1	1.6	10.6	20	10–30
	N259Q	0.017 ± 0.002	0.07	16.3	360	90–360
His263 (-2)	H263R	0.20 ± 0.01	0.8	17	90	40–240
	H263A	0.14 ± 0.03	0.6	21	240	90–360
Asp467 (-1/-2)	D467A	0.34 ± 0.03	1.4	0.5	30	30–120
	D467E	2.5 ± 0.6	10.4	2.8	5	3–30
Leu574 (-2)	L574V	15 ± 3	65	1.9	1	1–5

* General acid/base. ** Assisting residue ^1^ SA_H_: specific hydrolytic activity: Conditions 250 μM LNB-*p*NP, 50 mM citrate / 50 mM phosphate buffer, pH 4.5 and 30 °C. ^2^ %H: percentage of hydrolase activity relative to wt. ^3^ %LNT yield (max): maximum yield (%) from donor substrate. Conditions: 2.5 mM of LNB-*p*NP, 200 mM of lactose, 50 mM citrate / 50 mM phosphate buffer, pH 4.5 and 30 °C. ^4^ Reaction time at which the yield of LNT is maximum. ^5^ Time interval in which %LNT is within ±5% than %LNTmax. ^6^ n.d.: not detected.

**Table 2:** Hydrolase activity on LNB-*p*NP and transglycosylation yields for LNT by LnbB double mutants.

MUTANT	SA_H_^1^ (s^−1^)	% H ^2^ (mut/wt)	% LNT ^3^ Yield (max)
W394F_Y419F	0.014 ± 0.009	0.06	4.7
W394F_N259A	0.0060 ± 0.0008	0.02	2.9
W394F_N259Q	0.0053 ± 0.0003	0.02	n.d
W394F_H263A	0.044 ± 0.003	0.2	5.2
H263R_Y419F	<0.01	<0.04	0.7
H263A_Y419F	0.05 ± 0.005	0.2	1
Q190L_Y419F	0.17 ± 0.01	0.7	9.7

^1^ SA_H_: specific hydrolytic activity: Conditions 250 μM LNB-*p*NP, 50 mM citrate/50 mM phosphate buffer, pH 4.5 and 30 °C. ^2^ %H: percentage of hydrolase activity relative to wt. ^3^ %LNT yield (max): maximum yield (%) from donor. Conditions: 2.5 mM of LNB-*p*NP, 200 mM of lactose, 50 mM citrate/50 mM phosphate buffer, pH 4.5 and 30 °C.

**Table 3:** Specific activities (SA_H_, SA_T_) for transglycosylating LnbB mutants.

MUTANT	SA_H_^1^ (s^−1^)	SA_T_^2^ (s^−1^)	App.T/H Ratio ^3^
wt	27.7 ± 1.54	27.96 ± 2.63	1.0
W394F	0.069 ± 0.003	0.098 ± 0.0001	1.4
W394A	0.024 ± 0.004	0.018 ± 0.002	0.8
H263R	0.169 ± 0.003	0.22 ± 0.02	1.3
H263A	0.135 ± 0.001	0.13 ± 0.02	0.9
N259Q	0.017 ± 0.001	0.023 ± 0.005	1.3
Q190L	0.135 ± 0.003	0.122 ± 0.004	0.9

^1^ SA_H_ at 250 μM LNB-*p*NP. ^2^ SA_T_ at 250 μM LNB-*p*NP, 200 mM lactose. ^3^ Apparent T/H ratio as SA_T_/ SA_H_ ratio in the presence and absence of lactose acceptor determined from the rates of *p*NP release. Conditions: 50 mM citrate/50 mM phosphate buffer, pH 4.5 and 30 °C.

**Table 4:** Kinetic parameters (donor substrate) of LnbB-catalyzed hydrolysis and transglycosylation reactions.

MUTANT	Hydrolysis Mode			Transglycosylation Mode			
	*k*_cat_ (s^−1^)	*K*_M_ (μM)	*k*_cat_/*K*_M_ (M^−1^ · s^−1^)	*k*_cat_ (s^−1^)	*K*_M_ (μM)	*k*_cat_/*K*_M_ (M^−1^ · s^−1^)	T/H (*k*_cat_/*K*_M_) ^1^
WT	80 ± 8	520 ± 120	1.53·10^5^	90 ± 13	750 ± 200	1.20·10^5^	0.8
W394F	0.186 ± 0.008	260 ± 30	7.18·10^2^	0.26 ± 0.02	240 ± 52	1.09·10^3^	1.5
W394A	0.041 ± 0.003	87 ± 22	4.58·10^2^	0.06 ± 0.01	530 ± 190	1.19·10^2^	0.3
H263R	1.0 ± 0.2	780 ± 310	1.29·10^3^	1.0 ± 0.2	720 ± 300	1.44·10^3^	1.1
H263A	0.67 ± 0.03	670 ± 60	9.82·10^2^	1.08 ± 0.18	1280 ± 320	8.93·10^2^	0.9
N259Q	0.034 ± 0.002	235 ± 42	1.45·10^2^	0.07 ± 0.01	490 ± 150	1.50·10^2^	1.0
Q190L	0.201 ± 0.009	34 ± 7	5.93·10^3^	0.41 ± 0.02	78 ± 14	5.24·10^3^	0.9

^1^ Apparent T/H ratio calculated as the ratio of *k*_cat_/*K*_M_ values. Conditions: hydrolysis 10–1000 μM LNB-*p*NP, transglycosylation 10-1000 μM LNB-*p*NP, 200 mM lactose, in 50 mM citrate/50 mM phosphate buffer, pH 4.5 and 30 °C.

## Data Availability

The data that support the findings of this study are available from the corresponding author upon reasonable request.

## Supplementary Materials

The following are available online at https://www.mdpi.com/1422-0067/22/6/3230/s1.
