# Supplementary material for: Transglycosylation Activity of Engineered Bifidobacterium Lacto-N-Biosidase Mutants at Donor Subsites for Lacto-N-Tetraose Synthesis
Source: Int J Mol Sci. 2021 Mar 22;22(6):3230. doi: 10.3390/ijms22063230 (PMC8004761; doi:10.3390/ijms22063230)

SUPPLEMENTARY INFORMATION

Transglycosylation activity of engineered *Bifidobacterium* lacto-*N*-biosidase mutants at donor subsites for lacto-*N*-tetraose synthesis

Mireia Castejón-Vilatersana, Magda Faijes, Antoni Planas
Laboratory of Biochemistry, Institut Químic de Sarrià, University Ramon Llull, 08017 Barcelona, Spain

Table S1. Mutagenic primers.

Figure S1. Mechanism of GH20 enzymes acting by substrate-assisted catalysis.

Figure S2. Sequence alignment family GH20.

Figure S3. Initial rate of pNP release at increasing concentration of lactose acceptor.

Figure S4. Maximum yield of LNT formation (%LNT) *vs.* T/H ratio for the selected transglycosylating LnbB mutants.

Figure S5. Kinetics of pNP release catalyzed by LnbB mutants for donor substrate (LNB-pNP) in the absence and presence (200 mM) of lactose acceptor.

Figure S6. Thermal stability of LnbB wt and W394F mutant by thermal shift assay.

Table S1. Mutagenic primers.

**Mutation**	**Primer**	**Sequence**
**Q190L**	Fw	GTGCGCCTGTC**T**GATTAATATCTCTACGGATTGG
	Rv	GAGATATTAATC**A**GACAGGCGCACAGGGTCGCAC
**Q190H**	Fw	GTGCGCCTGTCA**C**ATTAATATCTCTACGGATTGG
	Rv	GATATTAAT**G**TGACAGGCGCACAGGGTCGC
**E216A**	Fw	GCTGCTGG**CG**ATGAAACTGAAACCGG
	Rv	GTTTCAT**CG**CCAGCAGCACGTAGTTC
**E216D**	Fw	GCTGCTGGA**T**ATGAAACTGAAACCGG
	Rv	GTTTCAT**A**TCCAGCAGCACGTAGTTC
**N259A**	Fw	CGGAAATT**GC**CAGTCCGGGTCACATGAACG
	Rv	CCCGGACTG**GC**AATTTCCGGGATCACATC
**N259Q**	Fw	CGGAAATTCA**GAG**TCCGGGTCACATGAACG
	Rv	CCCGGACT**CTG**AATTTCCGGGATCACATC
**H263A**	Fw	GTCCGGGT**GCG**ATGAACGTTTGGCTGGAAAATTATCC
	Rv	CGTTCAT**CGC**ACCCGGACTFTTAATTTCCGGG
**H263R**	Fw	CGGGTC**G**CATGAACGTTTGGCTGGAAAATTATCC
	Rv	CGTTCATG**C**GACCCGGACTGTTAATTTCCGGG
**D320A**	Fw	TGGGCGCG**GC**GAATACATGATTGGTACC
	Rv	CATGTATTCC**GC**CGCGCCCATGTGCCAATATTTCG
**D320E**	Fw	TGGGCGCGGGT**GAA**TACATGATTGGTACC
	Rv	CATGTATTC**TTC**CGCGCCCATGTGCCAATATTTCG
**E321A**	Fw	GCGCGGATG**CG**TACATGATTGGTACCTCG
	Rv	AATCATGTAC**GC**ATCCGCGCCCATGTGCCAATATTTCG
**W373F**	Fw	GCGCATCT**TC**AACGATGGTATTGTTAATACC
	Rv	CATCGTT**GA**AGATGCGCAGTTGTTTGC
**W394F**	Fw	GAATACT**TC**TACGGCGCCGGTCGTAAACCG
	Rv	GCGCCGTA**GA**AGTATTCGATAACGATGTCTTTG
**W394A**	Fw	CGAATAC**GC**GTACGGCGCCGGTCGTAAACC
	Rv	GCGCCGTAC**GC**GTATTCGATAACGATGTCTTTG
**W394K**	Fw	TTATCGAATAC**AAA**TACGGCGCCGGTCGTAAACC
	Rv	GCGCCGTA**TTT**GTATTCGATAACGATGTCTTTG
**W394E**	Fw	TCGAATAC**GAA**TACGGCGCCGGTCGTAAACC
	Rv	GCGCCGTATTCGTA**TTC**GATAACGATGTCTTTG
**W394Q**	Fw	CGAATAC**CA**GTACGGCGCCGGTCGTAAACC
	Rv	GGCGCCGTAC**TG**GTATTCGATAACGATGTCTTTG
**Y419F**	Fw	AGGCTCTGT**TC**TGGTCACGTTCGGCACAAG
	Rv	ACGTGACCA**GA**ACAGAGCCTGCGTTGCATTCATCAGG
**Y427F**	Fw	GCACAAGTGT**T**CAAAGTTAACGCAGCTCG
	Rv	GTTAACTTTG**A**ACACTTGTGCCGAACG
**W465F**	Fw	GTTTCGATTT**TC**CCGGATAGTTCCTATTTCC
	Rv	CTATCCGG**GA**AAATCGAAACTTTCGCAC
**D467A**	Fw	TTGGCCGG**CG**AGTTCCTATTTCCAGACC
	Rv	ATAGGAACT**CG**CCGGCCAAATCGAAACTTTCG
**D467E**	Fw	GATTTGGCCGGA**A**AGTTCCTATTTC
	Rv	GAAATAGGAACT**T**TCCGGCCAAATCGAAAC
**L574V**	Fw	CAAAACAT**G**TGGATGTCGTTACCC
	Rv	ACGACATCCA**C**ATGTTTTGAACCGG
**Q190L_Y419F[1]**	Fw	GTGCGCCTGTC**T**GATTAATATCTCTACGGATTGG
	Rv	GAGATATTAATC**A**GACAGGCGCACAGGGTCGCAC
**H263A_Y419F[1]**	Fw	GTCCGGGT**GCG**ATGAACGTTTGGCTGGAAAATTATCC
	Rv	CGTTCAT**CGC**ACCCGGACTFTTAATTTCCGGG
**H263R_Y419F[1]**	Fw	CGGGTC**G**CATGAACGTTTGGCTGGAAAATTATCC
	Rv	CGTTCATG**C**GACCCGGACTGTTAATTTCCGGG
**W394F_H263A[2]**	Fw	GTCCGGGT**GCG**ATGAACGTTTGGCTGGAAAATTATCC
	Rv	CGTTCAT**CGC**ACCCGGACTFTTAATTTCCGGG

**W394F_Y419F**[1]	Fw<br>Rv	GAATACT**TC**TACGGCGCCGGTCGTAAACCG<br>GCGCCGTA**GA**AGTATTCGATAACGATGTCTTTG
**W394F_ N259A**[2]	Fw<br>Rv	CGGAAATT**GC**CAGTCCGGGTCACATGAACG<br>CCCGGACTG**GC**AATTTCCGGGATCACATC
**W394F_ N259Q**[2]	Fw<br>Rv	CGGAAATTCA**GAG**TCCGGGTCACATGAACG<br>CCCGGACT**CTG**AATTTCCGGGATCACATC

[1] Using Y419F plasmid as a template

[2] Using W394F plasmid as a template

Figure S1. Mechanism of GH20 enzymes acting by substrate-assisted catalysis.

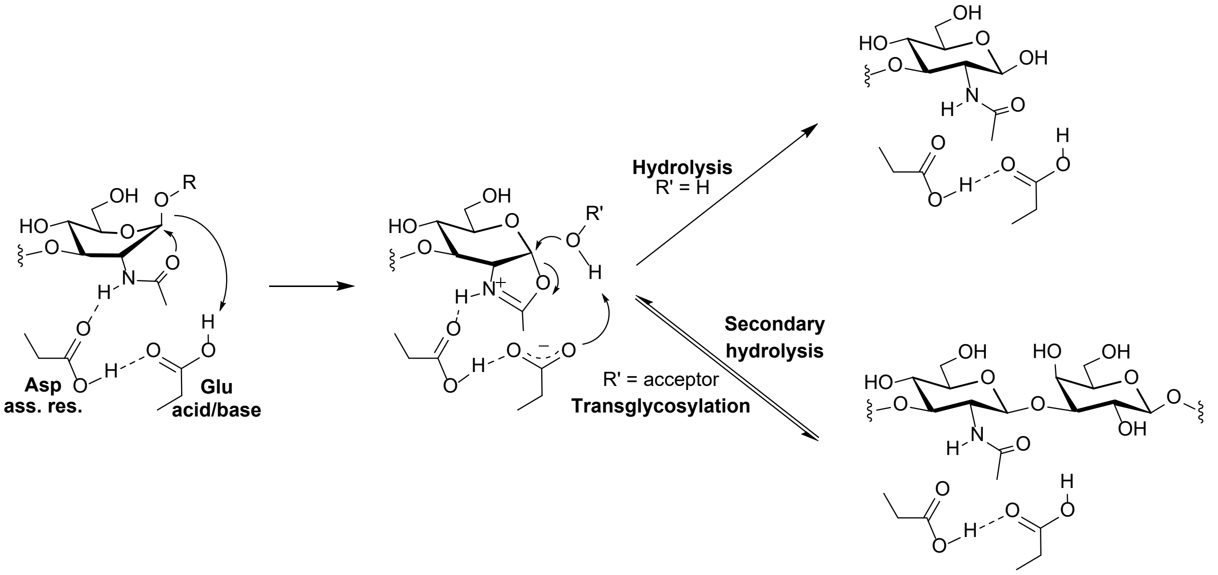

Upon substrate binding, the assisting Asp hydrogen bonds with the 2-acetamido group of the substrate and the general acid residue (Glu), which protonates the glycosidic oxygen and facilitates leaving group departure. In a concerted fashion, a nucleophilic attach of the 2-acetamido group of the substrate, now activated by the assisting residue, leads to the formation of the oxazoline/oxazolonium intermediate. In the second step, the intermediate is attacked by a nucleophilic water molecule assisted by the conjugate base of the general acid residue leading to the hydrolysis product. Transglycosylation occurs when an acceptor other than water binds to the acceptor subsites and attacks the intermediate assisted by the general base to give the transglycosylation product. Since the product is a substrate for the hydrolase activity of the enzyme, the transglycosylation product is susceptible to hydrolysis (secondary hydrolysis).

Figure S2. Sequence alignment family GH20.

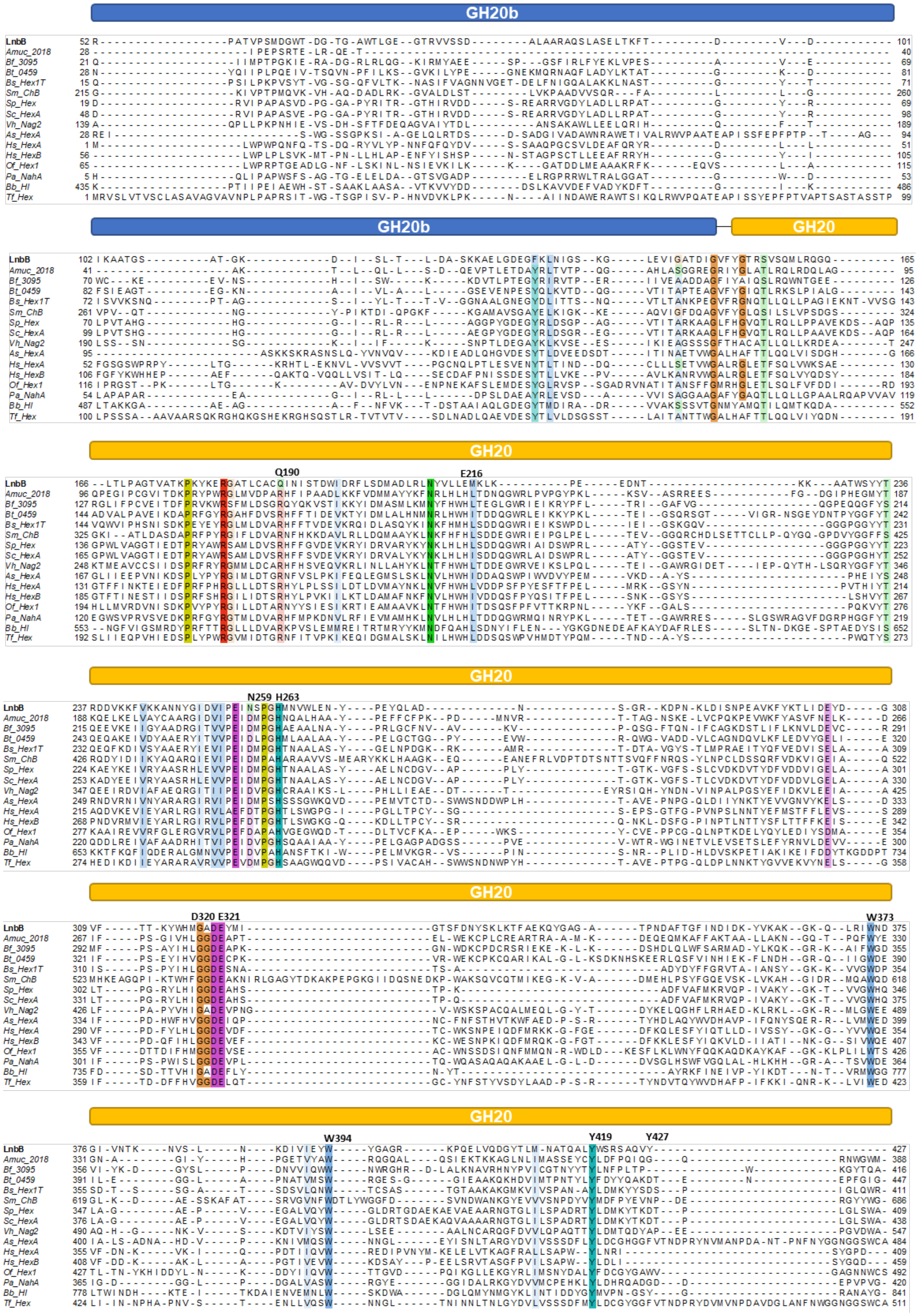

Figure S2.cont.

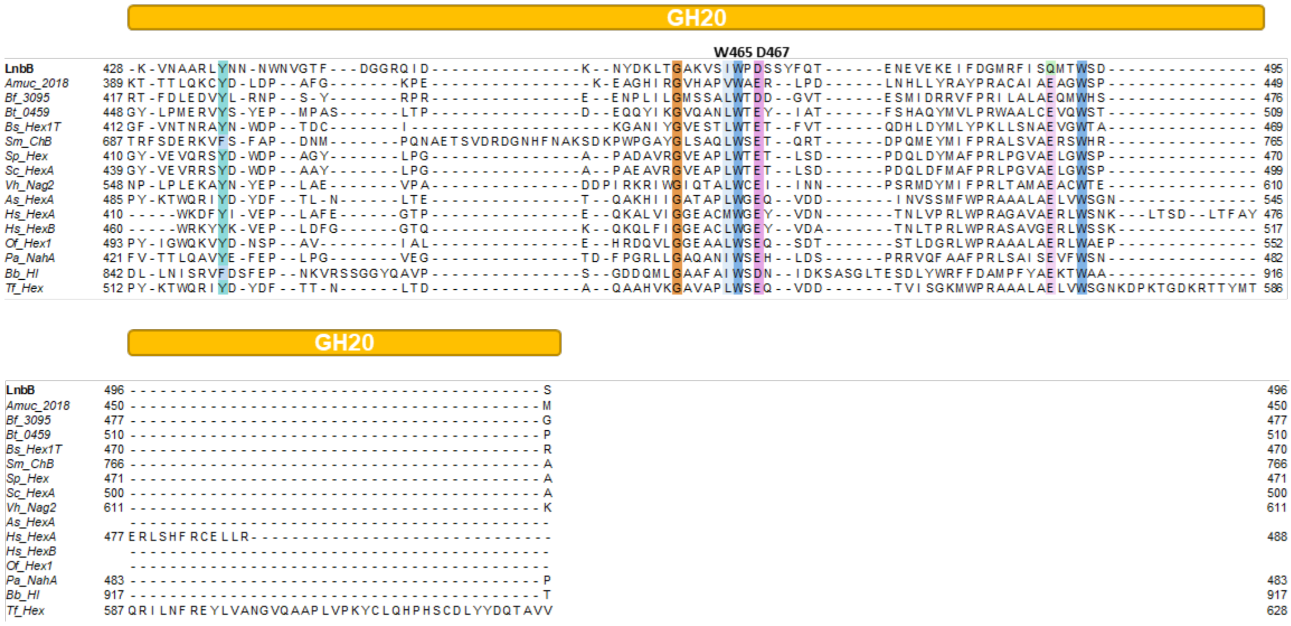

Proteins in the alignment (in alphabetical order): **Amuc2018** (ß-N-acetylhexosaminidase from *Akkermansia muciniphila* ATCC BAA-835, B2UP57), **As_HexA** (ß-N-acetylhexosaminidase from *Aspergillus oryzae* CCF 1066, Q8J2T0), **Bb_HI** (ß-N-acetylhexosaminidase from *Bifidobacterium bifidum* JCM 1254, D4QAP4), **Bf_3095** (ß-N-acetylhexosaminidase from *Bacteroides fragilis*, Q5LAT3), **Bs_Hex1T** (ß-hexosaminidase from *Penicilium* Sp. TS12, D2kW09), **Bt_0459** (ß-N-acetylhexosaminidase from *Bacteroides thetaiotamicron* VPI-5482, Q8AAK5), **Hs_HexA** (ß-hexosaminidase A from *Homo Sapiens*, P06865), **Hs_HexB** (ß-hexosaminidase B from *Homo Sapiens*, P07686), **LnbB** (lacto-N-biosidase from *Bifidobacterium bifidum* JCM 1254, B3TLD6), **Of_Hex1** (ß-N-acetylhexosaminidase from *Ostrinia furnacalis*, Q06GJ0), **Pa_NahA** (ß-N-acetylhexosaminidase from *Paenarthrobacter aurescensc* TC1, A1RBZ5), **Sc_HexA** (ß-N-acetylhexosaminidase from *Streptomices coelicolor* A3(2), Q9L068), **Sm_ChB** (chitobiase from *Serratia marcescens* 2170, Q54468), **Sp_Hex** (ß-N-acetylglucosaminidase from *Streptomyces plicatus*, O85361), **Tf_Hex** (ß-N-acetylhexosaminidase from *Talaromyces flavus*, AEQ33603.1), **Vh_Nag2** (ß-N-acetylglucosaminidase from *Vibrio harveyi* 650, D9ISE0). Alignment was performed with TcoffeeWS. Conserved positions are colored according to Clustal parameters.

Figure S3. Initial rate of pNP release at increasing concentration of lactose acceptor. Conditions: 250 µM LNB-pNP, 0-600 mM lactose, 50 mM citrate / 50 mM phosphate buffer, pH 4.5 and 30 °C.

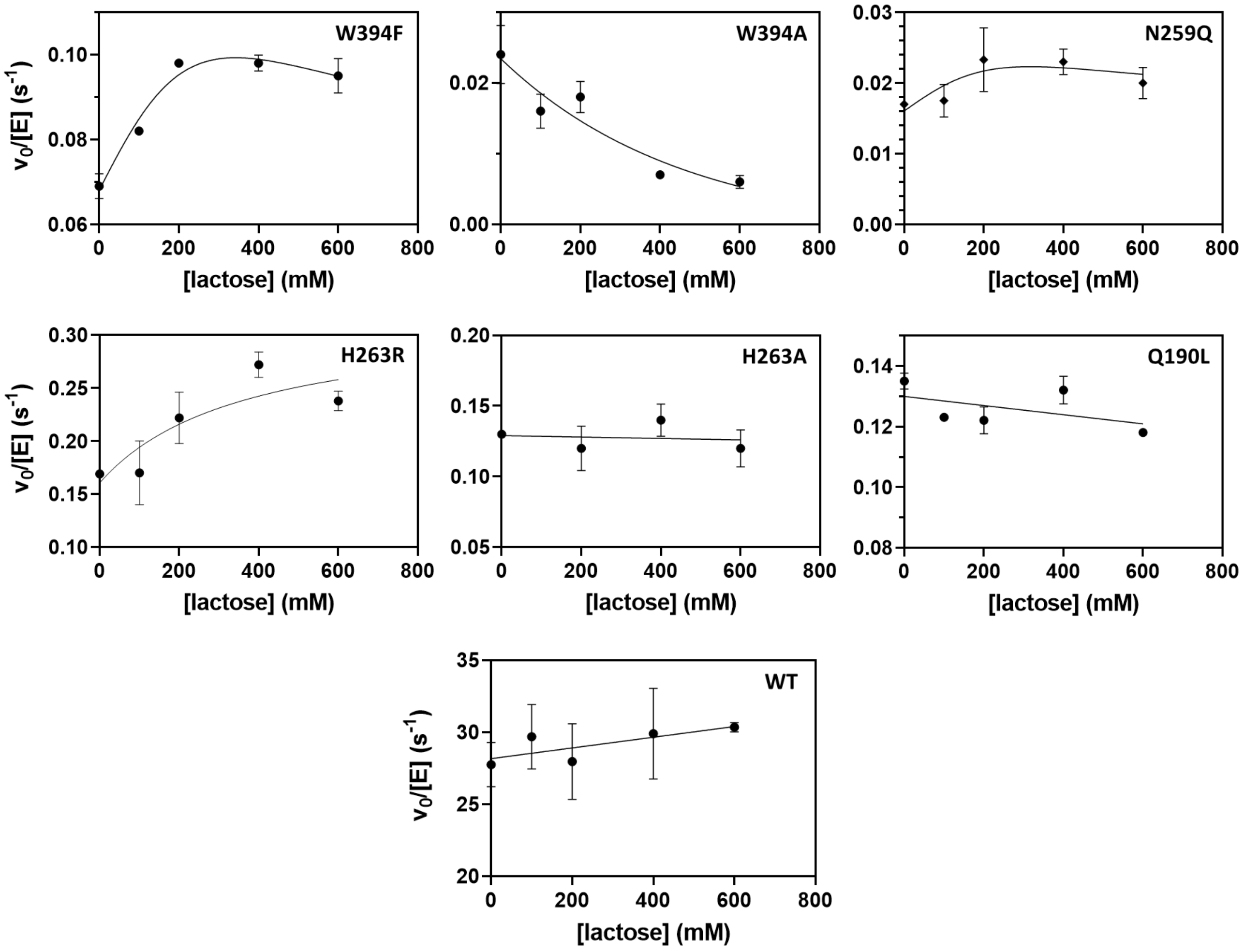

Figure S4. Maximum yield of LNT formation (%LNT) *vs.* apparent T/H (k_{cat}/K_m) ratio for the selected transglycosylating LnbB mutants.

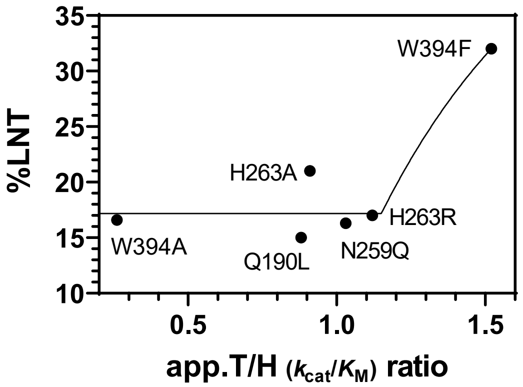

Figure S5. Kinetics of pNP release catalyzed by LnbB mutants for donor substrate (LNB-pNP) in the absence and presence (200 mM) of lactose acceptor. Conditions: 50 mM citrate / 50 mM phosphate buffer, pH 4.5 and 30 °C.

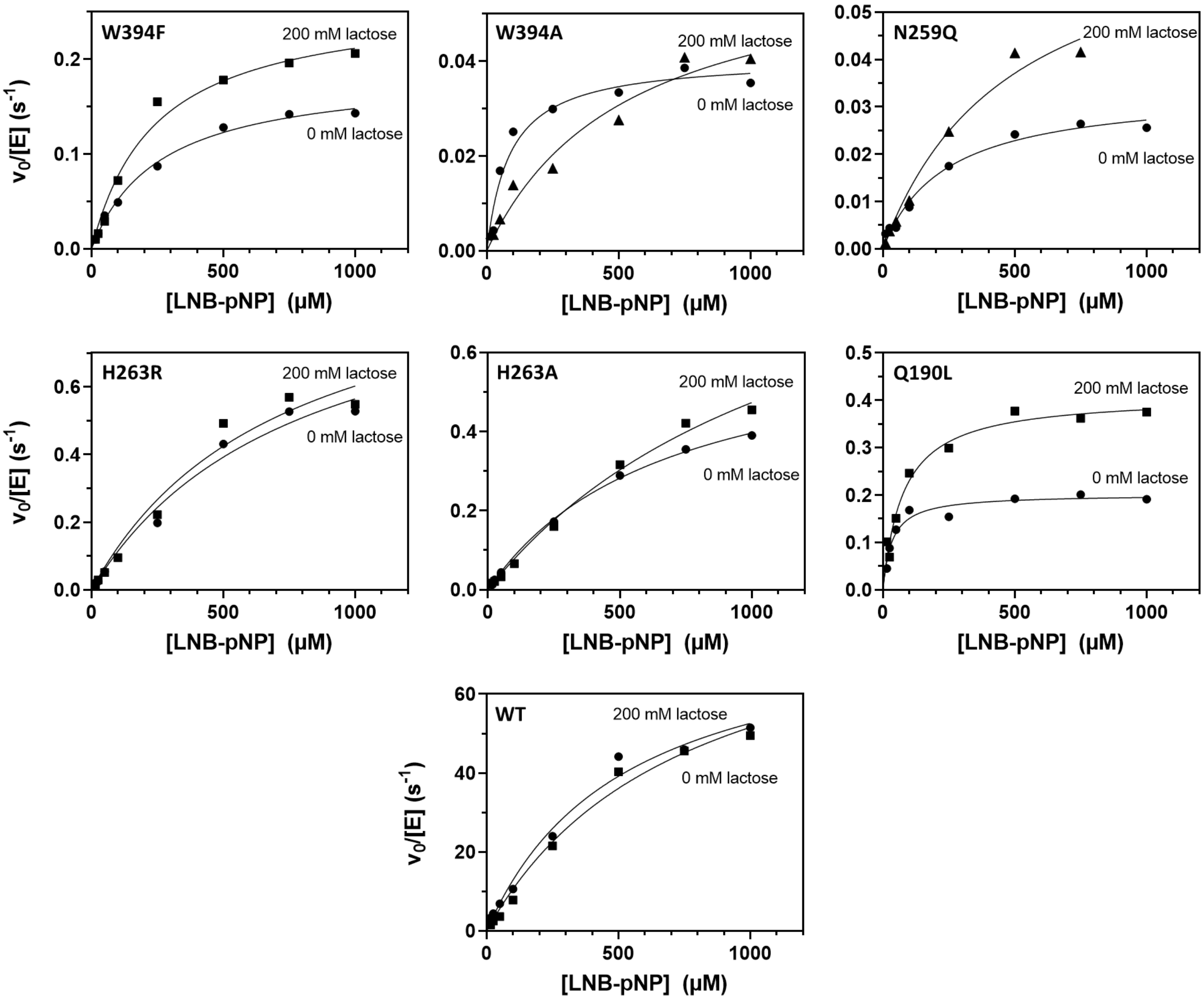

Figure S6. Thermal stability of LnbB wt and W394F mutant by thermal shift assay using Sypro Orange dye and fluorescence monitoring (λex 483 nm, λem 560 nm).

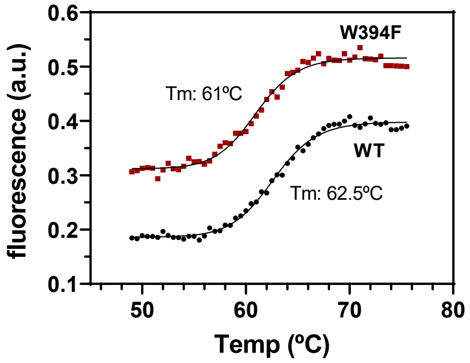

Supplement: Supplementary file 1 [file ijms-22-03230-s001.pdf]
